# Supplementary material for: Efficient Activation of Peroxymonosulfate by Cobalt Supported Used Resin Based Carbon Ball Catalyst for the Degradation of Ibuprofen
Source: Materials (Basel). 2022 Jul 18;15(14):5003. doi: 10.3390/ma15145003 (PMC9321845; doi:10.3390/ma15145003)
Supplement: Supplementary file 1 [file materials-15-05003-s001.zip › materials-1795695-supplementary.pdf]

Efficient activation of perxymonosulfate by cobalt supported used resin based carbon ball catalyst for the degradation of ibuprofen

Guangzhen Zhou [a], Yanhua Xu [a], Xiao Zhang [a], Yongjun Sun [b], Cheng Wang [a,]* and Peng Yu [a],

[a] School of Environmental Science and Engineering, Nanjing Tech University, Nanjing, 211816, China
[b] College of Urban Construction, Nanjing Tech University, Nanjing, 211816, China

*Corresponding Author: Cheng Wang
Tel.: +86-18512525302. Fax: +86-025-58139652
E-mail: yz_wc19890901@163.com.

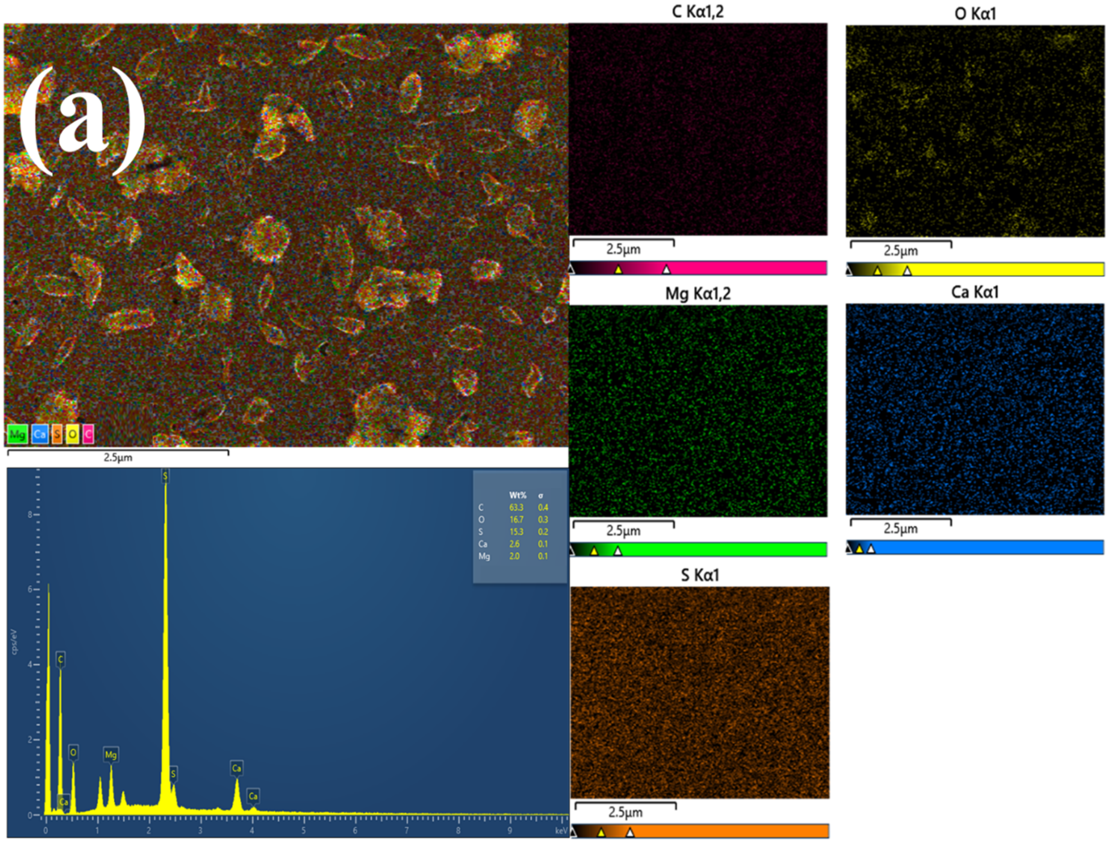

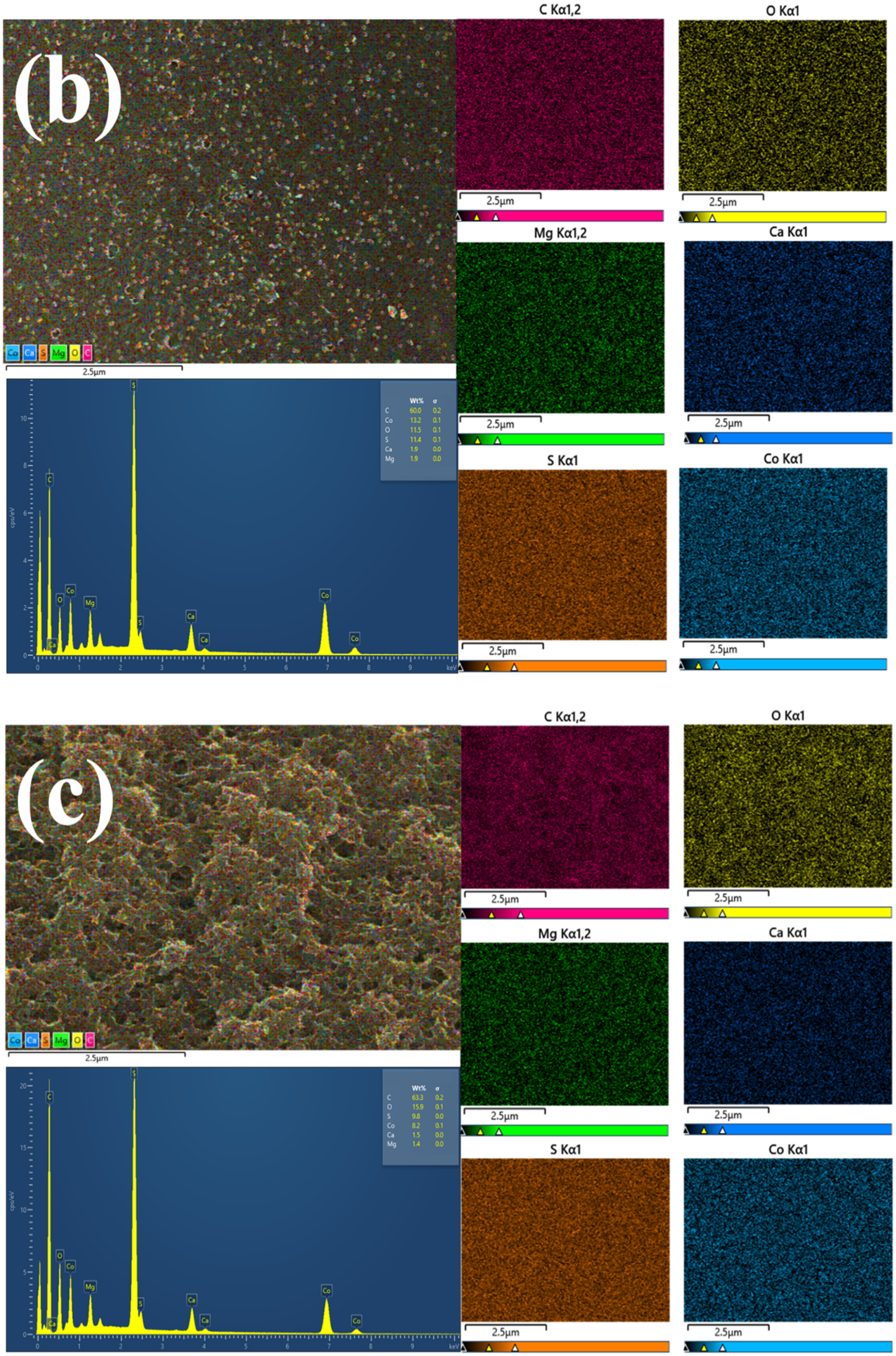

Figure S1. EDS element layered image of material, carbonization of waste resin (a), the surface and inside of carbonized cobalt doped waste resin respectively(b, c).

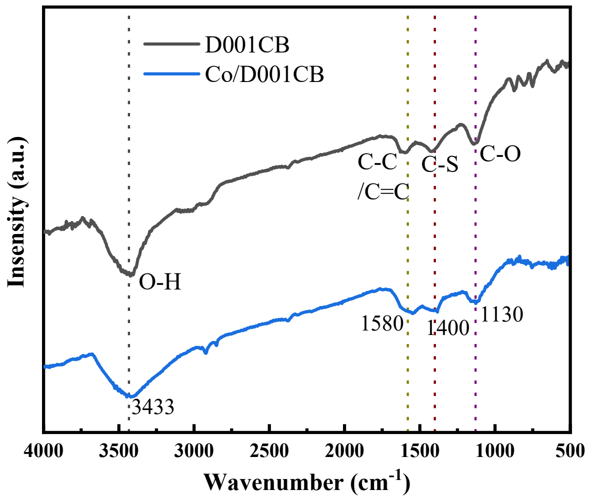

Figure S2. FTIR spectrums of carbonized used resin and cobalt doped used resin.

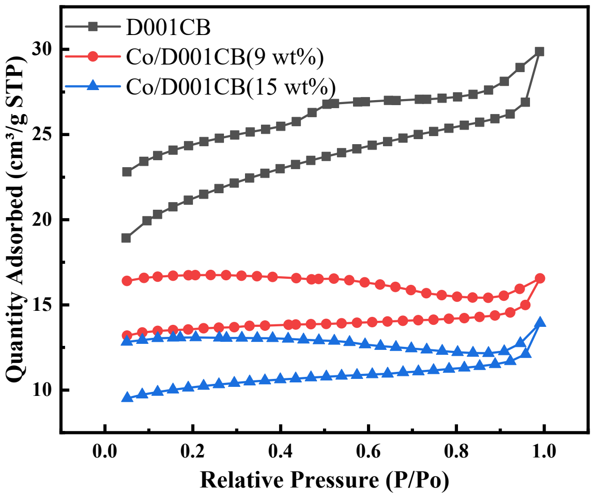

Figure S3. Nitrogen adsorption desorption curves of carbonized used resin and cobalt doped used resin.
